# Uncovering the Novel Genetic Determinants of Primary Congenital Glaucoma in Pakistani Families

**DOI:** 10.1155/humu/1806353

**Published:** 2026-09-18

**Authors:** Hamza Khan, Sadia Bukhari, Nauman Ahmad, Israr Ahmed Bhutto, Yousaf Jamal Mahsood, Mohsin Iqbal Haroon, Muhammad Moeez Uddin, Amal Shabir Memon, Atta Ur Rehman, Shah Zainab, Asad Munir, Syeda Hafiza Benish Ali, Muhammad Ajmal, Raheel Qamar, Jibril Hirbo, Maleeha Azam, Humaira Ayub

**Affiliations:** ^1^ Department of Biological and Health Sciences, Pak Austria Fachhochschule: Institute of Applied Science and Technology, Haripur, Pakistan; ^2^ Al-Ibrahim Eye Hospital, Karachi, Pakistan; ^3^ Arsalan Eye Clinic, Multan, Pakistan; ^4^ Department of Ophthalmology, Jinnah Postgraduate Medical Center, Karachi, Pakistan; ^5^ Department of Ophthalmology, Khyber Girls Medical College, Hayatabad Medical Complex, Peshawar, Pakistan, hmcpeshawar.com.pk; ^6^ Glaucoma Department, Sindh Institute of Ophthalmology and Visual Sciences (SIOVS), Hyderabad, Pakistan; ^7^ Prevention of Blindness (POB) Trust Eye Hospital, Karachi, Pakistan; ^8^ Department of Zoology, Faculty of Biological and Health Sciences, Hazara University, Mansehra, Pakistan, hu.edu.pk; ^9^ Department of Biosciences, COMSATS University Islamabad, Islamabad, Pakistan, comsats.edu.pk; ^10^ Department of Medicine, Vanderbilt University Medical Center, Nashville, Tennessee, USA, vanderbilt.edu

**Keywords:** congenital glaucoma, *CYP1B1*, genetic variations, linkage analysis, novel locus, whole-exome sequencing

## Abstract

Primary congenital glaucoma (PCG) is an early‐onset eye disorder, particularly prevalent in populations with high consanguinity rates. In Pakistan, approximately 63% of marriages are reported to be consanguineous, contributing to an increased burden of autosomal‐recessive disorders. The genetic basis of PCG in the highly consanguineous Pakistani population is largely unexplored. This study was conducted to broaden the genetic landscape of PCG in families of Pakistani origin. PCG families identified through clinical characterization were recruited in the study. All probands were initially investigated for *CYP1B1* mutations by targeted Sanger sequencing, followed by segregation analysis of the identified variants. Moreover, one family (PCG02) not linked to *CYP1B1* was further subjected to a 250K SNP microarray and whole‐exome sequencing (WES). The study participants comprised nine large, unrelated consanguineous and three PCG trio families collected from Pakistan. Nine identified variants were recurrent, whereas one previously unreported variant, *CYP1B1* (NM_000104.4: c.1390T>C; p.Ser464Pro), was not found in any of the online databases such as gnomAD, ClinVar, and dbSNP, and was predicted to be deleterious by all the pathogenicity predicting tools. Furthermore, a plausible 2‐Mb novel locus was identified on Chromosome 7q34 (139,681,371‐141,507,822) (GRCh38/hg38) in family PCG02. The current study expands the mutation spectrum of *CYP1B1* in the Pakistani population and highlights the genetic heterogeneity of the disease in this population.

## 1. Introduction

Primary congenital glaucoma (PCG; OMIM 231300) is a genetically heterogenous autosomal recessive disorder of the eye, characterized by developmental defects in the trabecular meshwork (TM) and anterior chamber angle, appearing in neonates (onset at birth to less than 1 month) or infants (onset from 1 to 24 months) [1]. The maldevelopment of the anterior chamber angle interferes with the aqueous humor drainage, which subsequently results in elevation of intraocular pressure (IOP > 21 mm of Hg) [2]. Consequently, these changes further lead to buphthalmos (ocular enlargement), optic nerve cupping, megalocornea (enlarged corneal diameter), edema and opacification of cornea, epiphora (excessive watering of the eyes), photophobia, and ultimately, blindness [3, 4]. The ratio of congenital glaucoma is 1/2000 live births in the Middle East, whereas the ratio in western countries is 1/10,000 [5]. Although a comprehensive PCG prevalence report is still lacking in Pakistan, studies suggested the higher percentage of consanguineous marriages is attributed to higher ratio of genetic disorders in Middle Eastern countries like Saudi Arabia [6], and this ratio is even higher in South Asian countries including Pakistan [7]. According to a study conducted by British Infantile and Childhood Glaucoma (BIG) in United Kingdom and Republic of Ireland, the incidence of PCG is almost nine times higher in Pakistani children than among the white Caucasian children [8].

Genetic studies demonstrated the linkage of PCG to the GLC3A (glaucoma 3, primary congenital, A) loci mapped to 2p21 that harbors cytochrome P450 family 1 subfamily B member 1 (*CYP1B1*; OMIM 601771) [9], GLC3D (glaucoma 3, primary congenital, D) mapped to 14q24 [10] on which latent transforming growth factor beta binding protein 2 (*LTBP2*; OMIM 602091) is present [11], and GLC3E (glaucoma 3, primary congenital, E) at 9p21 harboring tunica interna endothelial cell kinase (*TEK*; OMIM 600221) [12]. Genes in the GLCB‐C regions remain unidentified [13]. Another gene myocillin (MYOC; OMIM 601652) has been suggested to be associated with PCG [14] as well, which is present at GLC1A (glaucoma 1, open angle, A) locus mapped to 1q24.3 [14]. Mutant *CYP1B1* is the most prevalent PCG causative gene globally as well as in Pakistan [4] followed by *LTPB2* [11]. The most common *CYP1B1-*associated PCG mutation among Pakistani population is c.1169G>A; p.Arg390His [5] and is considered a founder mutation in the Pakistani population [15]. More than 39 mutations in *CYP1B1* and 12 mutations in *LTBP2* have also been identified to be causative of glaucoma in Pakistani families [16].

The multiple mutations already identified, increased genetic heterogeneity, and higher rates of PCG in the Pakistani population accentuate the need for an in‐depth understanding of the underlying molecular mechanisms. The purpose of the current study was to genetically characterize the consanguineous Pakistani PCG families and to further explore novel genetic factors associated with PCG.

## 2. Materials and Methods

### 2.1. Clinical Assessment, Sample Collection, and DNA Isolation

This study was approved by the Research Ethical Committee (REC) of Pak‐Austria Fachhochschule; Institute of Applied Science and Technology (Approval No. PAF‐IAST/2021/08). Informed written consent was taken from all probands (consent taken from parents in case of patients under 18 years old) and recorded.

Detailed clinical analysis was done for all the members of each family. These tests included fundoscopy, tonometry, gonioscopy, and visual field analysis for all patients, and B‐scan ocular ultrasound was performed whenever required. Moreover, buphthalmos, watering and frequency of pain were also noted. The patients were clinically examined at Al‐Ibrahim Eye Hospital, Karachi and Hayatabad Medical Complex, Peshawar.

A total of 5 mL of venous blood was obtained from each participant in EDTA tubes. Extraction of genomic DNA was done using the conventional phenol‐chloroform method.

### 2.2. Exclusion Mapping of *CYP1B1*

Primers of coding exons and exon intron boundaries of *CYP1B1* were designed using Primer3 Input (https://primer3.ut.ee/) (primer sequence can be provided on demand).

The amplicons of the probands were subjected to Sanger sequencing (BigDye Terminator V3.1, Applied Biosystems, Foster City, California) on SeqStudio genetic analyzer (Applied Biosystems, Foster City, California) at the in‐house Genomics facility at PAF‐IAST. The sequences were further analyzed using UCSC genome browser (University of California, Santa Cruz) BLAT tool (https://genome.ucsc.edu/cgi-bin/hgBlat?command=start).

### 2.3. In‐Silico Analysis of Identified Variants

The functional effects of the identified variants were predicted by the in‐silico pathogenicity predicting programs: sorting intolerant from tolerant (SIFT) (https://sift.bii.a-star.edu.sg) to predict effect of variants on protein function, polymorphism phenotyping Version 2.0 (Polyphen‐2) (http://genetics.bwh.harvard.edu/pph2/) to predict the impact of amino acid substitution on protein structure and function, MutationTaster (https://www.mutationtaster.org/) to predict impact of mutation on protein, and Varsome (https://varsome.com/) for variant interpretation and classification. The multiple sequence alignment of the *CYP1B1* was done using Clustal Omega (https://www.ebi.ac.uk/jdispatcher/msa/clustalo) to observe the conservation of amino acid Serine at 464 residue. To predict the biochemical changes in the protein structure as a result of the variant, HOPE software (https://www3.cmbi.umcn.nl/hope/) was used. I‐Mutantv2.0 software (https://folding.biofold.org/i-mutant/i-mutant2.0.html) was used to predict the changes in protein stability based on the Gibbs free energy ($\Delta \Delta \text{G}=\Delta {{\text{G}}_{f}}^{wt}-\Delta {{\text{G}}_{f}}^{\text{mut}}$) as a result of the variation.

### 2.4. Three‐Dimensional Protein Model Prediction for Variant p.Ser464Pro

InterPro (https://www.ebi.ac.uk/interpro/) online database was used to identify the functional domains of full‐length CYP1B1 protein to identify the location of Ser464 residue. The wild‐type human CYP1B1 protein sequence (UniProtKB accession Q16678) and its corresponding structural model were obtained from the UniProt (https://www.uniprot.org/) database. An AlphaFold‐predicted model of the wild‐type CYP1B1 was retrieved from the UniProt/AlphaFold repository in PDB format. This provided a reference 3D conformation of the native protein for comparison with its mutant form.

The mutant sequence was used as input to AlphaFold Server (https://alphafoldserver.com/welcome) for structure prediction. Specifically, the ColabFold implementation of AlphaFold was employed to compute the 3D structure of the Ser464Pro variant. Upon completion of the prediction run, the Ser464Pro mutant model was obtained in PDB format. Both the wild‐type and mutant (Ser464Pro) CYP1B1 structures were visualized and analyzed using PyMOL (https://www.pymol.com/). Both models were loaded into PyMOL and aligned by backbone superposition with the built‐in alignment tools. Structural alignment was evaluated by calculating the root‐mean‐square deviation (RMSD) between corresponding atoms and by visual inspection of the overlay. In particular, the region surrounding Residue 464 was examined in detail to identify any mutation‐induced conformational differences. Secondary structure elements and overall folding topology of the wild‐type and mutant proteins were compared to assess the impact of the Ser464Pro mutation on the CYP1B1 structure.

### 2.5. Linkage Analysis and Exome Sequencing for Family PCG02

The family PCG02 was further subjected to the linkage analysis. Affymetrix 250K SNP microarray analysis was done for both affected individuals of the family, and obtained data were further subjected to homozygosity mapping. Homozygous regions larger than 1 Mb were further subjected to CA‐repeat marker analysis to find the true homozygous region (Table S1). Highly polymorphic microsatellite markers with reported heterozygosity values greater than 70% were selected for the analysis.

Further exome sequencing for both patients was done from Life Technologies (Carlsbad, California). Exome sequencing revealed thousands of variants and therefore the number was narrowed down by using various filters. The variants shared by both individuals were included for further analysis. The variants that were detected in less than 10 reads were removed. Exclusion of synonymous and noncoding variants resulted in a few hundred remaining variants. The variants with a population frequency of less than or equal to 0.5% in the dbSNP database were retained. Moreover, filtering for homozygous variants was conducted by retaining variants that were present in more than 80% of variant calls. The homozygous exome variants with a Grantham score higher than 80 or a phyloP score of more than 2.0 were retained. No variants were obtained in the novel locus after using these filters; therefore, less stringent conditions for filtration were applied on the data; hence, few genes lied below our cutoff value of Grantham and phyloP. Integrative genomics viewer (IGV) software was used to detect missing reads present in the sequence data. Candidate variants obtained after filtration were subjected to segregation analysis by Sanger sequencing. In‐silico analysis (mentioned previously) was used to predict the functional effects of filtered exome variants.

## 3. Results

Nine families belonging to Sindh, Khyber Pakhtunkhwa (KPK), Punjab, and Baluchistan, consisting of more than one affected individual, and three trio families of PCG consisting of only one affected individual along with parents were recruited in the study. A 2 MB plausible novel locus at Chromosome 7q34 (139,681,371–141,507,822) was identified in one PCG family. All the other families and trio familial cases were linked to *CYP1B1*, with one family harboring previously unreported variant.

### 3.1. Clinical Manifestation Among PCG Families and Trios Cases

PCG02 consisted of two affected members (both males) and symptoms appeared in both affected individuals in the first 3 months of their life and were progressive in nature, eventually resulting in complete blindness. The affected individuals had buphthalmia, raised IOP, bilateral nystagmus, completely avascularized, and opaque corneas (Table 1, Figure S1). Routine ophthalmic tests were not possible for the affected individuals because corneas were highly sensitive to touch. The IOP measurement was conducted carefully with air puff tonometry, and in both individuals, IOP was found to be > 40 mmHg. Due to the corneal opacity the patient′s optic nerve and the fundus could not be visualized. The patients also had horizontal breaks in the Descemet′s membrane (Haab′s striae). Trabeculectomy had been done for one affected individual (II:IV) to lower the IOP and pain in the eyes. No signs of PCG were observed in all the other unaffected siblings and parents.

**Table 1:** Clinical characters of the probands with PCG recruited in the study.

S. no.	Family/trios ID	Patient ID	Age of onset of disease	Age (years) at time of recruitment	IOP at presentation (mmHg)	Max IOP recorded	CDR	Corneal diameter (mm)	Visual acuity	Other findings
1	PCG02	II:IV	3 months	13	NA/NA	38/40	NV/NV	Increased	NPL/NPL	B/L Bu, B/L Mc, B/L CO, B/L CH, Nys, CE
2	PCG03	III:III	By birth	24	20/22	NA	NV/NV	15/15	NPL/NPL	B/L Bu, B/L Mc, B/L CO, B/L CH
3	PCG04	V:II	By birth	7	29/28	40/40	NV/NV	15/15	NPL/NPL	B/L Bu, B/L Mc, B/L CO, B/L CH, B/L HS
4	PCG05	III:II	By birth	16	12/12	NA	NV/NV	15/15	3/60/NPL	B/L Bu, B/L Mc, B/L CO, B/L CH, U/L HS
5	PCG06	IV:II	By birth	22	16/‐	40/‐	1.0/‐	Increased	6/60/NPL	U/L Bu, U/L Mc, LBE
6	PCG07	IV:II	By birth	6	17/26	NA	0.5/0.4	12.5/12	6/36/6/19	B/L Bu, B/L CE
7	PCG08	III:III	By birth	21	18/20	35/42	1.0/NV	15/15	HM/6/60	B/L Bu, B/L Mc, U/L CH
8	PCG11	IV:II	By birth	0.6	10/12	NA	NV/NV	Increased	NFL/NFL	B/L Bu, B/L Mc, U/L CO, U/L CH
9	PCG13	IV:VI	By birth	1.4	16/14	42/44	0.3/NV	14/14	PL/NPL	B/L Bu, B/L Mc, U/L CO, U/L CH, U/L Cat, CE
10	PCGT01	II:I	By birth	4	17/14	NA	0.3/0.4	13/13.5	6/12/6/12	B/L Bu, B/L Mc, B/L CH, CE
11	PCGT02	II:I	By birth	8	10/12	28/34	NV/0.4	13.5/13	HM/6/19	B/L Bu, B/L Mc, U/L CO, RD, Nys
12	PCGT03	II:I	By birth	6	8/10	NA	NV/NV	15/14.5	NPL/PL	B/L Bu, B/L CH, VH

Abbreviations: Bu, buphthalmos; B/L, bilateral; CDR, cup–disc ratio; CE, corneal edema; CH, corneal haze; CO, corneal opacity; FL, following light; HM, hand motion; HS, Haab′s striae; IOP, intraocular pressure; LBE, left blind eye; Mc, megalocornea; NA, not available; NFL, not following light; NPL, no perception of light; NV, not visible; Nys, nystagmus; PL, perception of light; RHE, right hard eye; U/L, unilateral; VH, vitreous hemorrhage.

Family PCG03 consisted of 10 affected members including seven females and three males. All the affected members showed severe manifestation of PCG. The proband (III:III) had no vision since birth, with corneal haze and opacity, and complaints of severe bilateral ocular pain thus resulting in enucleation in early life (Table 1).

PCG04 consisted of 11 affected individuals including eight females and three males (Figure S2). All the affected individuals had congenital onset of bilateral glaucoma and had common symptoms that included elevated IOP, bilateral buphthalmos, megalocornea, and poor visual acuity. The proband of PCG04 (IV:II) had maximum IOP recorded > 40 mmHg and had bilatelral corneal haze and opicity, and Haab′s striae in both eyes (Table 1) and same features were observed for other affected members. The family PCG05 consisted of three (3) affected individuals including one male and two females (Figure S2). The proband (III:II) showed severe PCG symptoms such as bilateral bupththalmos, megalocornea, corneal opacity, central corneal haze, and Haab′s striae (Table 1). The proband had multiple eye surgeries in childhood and had bilateral Ahmed glaucoma valve (AGV) implanted. The patient also had a failed corneal graft in the right eye. Depsite the early preventative measures, the Individual III:II had severe visual impairment with visual acuity of 3/60 in the right eye and no perception of light in the right eye. The other affected individual (II:IV) had same symptoms of PCG, and was completely blind. Family PCG06 consisted of two affected individuals (both males) (Figure S2). Both the affected individuals (IV:II and IV:III) had onset of PCG by birth. The proband (IV:II) had completely blind left phthisical eye, and the right eye had maximum IOP recorded of 40/‐, cup‐to‐disc ratio of 1.0/‐, and visual acuity of 6/60 (Table 1).

The family PCG07 consisted of three affected individuals including two males and one female (Figure S2). All the affected members of family had congenital onset of bilateral glaucoma. Surprisingly, the corneal diameter of all the patients were near to normal with no corneal opacity. Individuals IV:II (Table 1) and IV:IV underwent bilateral trabeculotomy and had normal CDR with preserved vision. Individual IV:VI did not undergo early surgical interventions, had severe vision loss and could perceive light only. Two of the normal siblings (Figure S2) of the affected individual (IV:VI) had aphasia. PCG08 consisted of four affected individuals including two males and two females (Figure S2).

All the affected members in PCG08 had onset of PCG by birth. The proband of PCG08 (III:III) had maximum IOP recorded as 35/42 mmHg, with corneal diameter of 15/15 mm, and visual acuity of hand motion (HM) in the right eye and 6/60 in the left eye. The proband had cup‐to‐disc ratio of 1.0 in the right eye, whereas the measurement was not possible in the right eye due to corneal haze (Table 1).

The other three affected members showed same PCG symptoms; however, Individual III:V had severe corneal opacity in the right eye with perception of light in the left eye, and Individual III:X had left phthisical eye at the age of 15 years and had perception of light in the left eye. Despite multiple surgeries in childhood, all the affected individuals of PCG08 had severe clinical manifestation of PCG.

PCG11 consisted of two affected individuals (both males) (Figure S2). The Proband IV:II had severe corneal opacity and could not follow light target (Table 1), whereas Patient IV:I had clear cornea with visual acuity of 6/15 in the right eye and 6/24 in the left eye. PCG13 consisted of six effected individuals including four males and two females (Figure S2). The proband (IV:IV) had maximum IOP recorded > 40 mmHg with mild corneal edema and perception of light in the right eye and no percetion of light in the left eye. Moreover, the left eye had corneal opacity and haze, and cataract (Table 1). The Pateints II:III, IV:V, and IV:VI underwent early surgical intervention to control IOP in both eyes, and had normal CDR, and perception of light in the right eyes. The left eyes of the patients (I:III, IV:V, and IV:VI) had mature catract, severe corneal opacity and haze, and no perception of light due to PCG. Variablity in corneal diameter (verticallty and horizantally) was also observed in Pateint IV:V having corneal diameter of 11/10 mm, whereas the other individuals (II:III, IV:V) had mean corneal diamter of 15 mm. Photophobia and Haab′s striae (right eye) were also observed in Pateint IV:V, whereas these symptoms were not observed in other pateints of the family. The Patients I:I and II:II did not undergo any surgical interventions and thus were completely blind.

In trios PCGT01, medical history showed that the early bilateral trabeculotomy in the patient (II:I) led to the control of IOP and preservation of vision, with normal CDR. The cornea was enlarged, but no corneal opacity was observed. However, mild corneal haze and edema were observed (Table 1). The parents of the individual (PCGT01) were not related. The PCGT02 patient (II:I) had bilateral buphthalmos and more severe symptoms of were observed in the right eye with retinal detachment, and visual acuity up to HM, whereas the left eye had clear cornea (Table 1). In trios PCGT03, the patient (II:I) had PCG by birth with severe clinical manifestation along with vitreous hemorrhage in the left eye (Table 1).

### 3.2. Genetic Analysis Results

#### 3.2.1. Identification of a Plausible 2‐MB Novel Homozygous Locus at Chromosome 7q34 in PCG02

**Figure 1:**
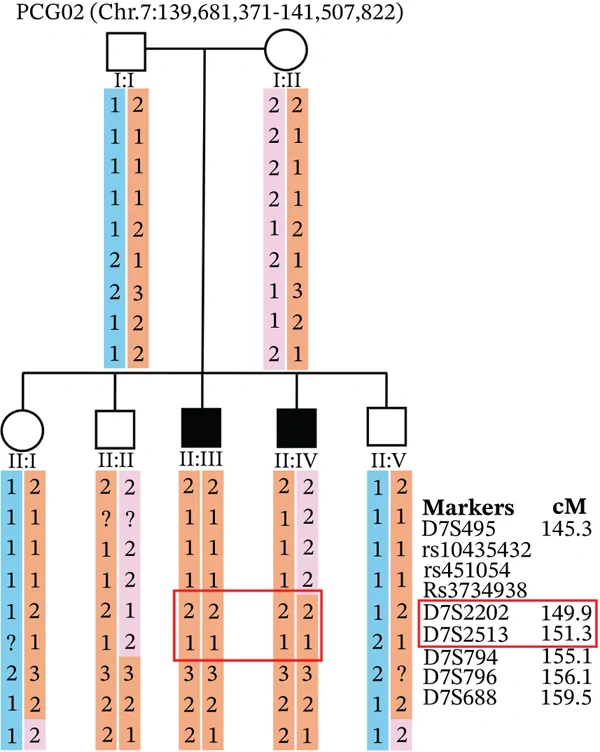
Homozygous region linked to PCG in two affected individuals (II:3 and II:4) of family. The novel locus was flanked by D7S2202 (139,681,371‐139,881,718 cM:149.9) and D7S2513 (141,307,418‐141,507,822 cM:151.3). The region highlighted red is the critical 2 MB interval.

For the *CYP1B1* unlinked family PCG02, homozygosity mapping revealed five homozygous regions on Chromosomes 3, 7, and 15 (Table S2; Figure S3). CA marker analysis confirmed the true homozygous region on Chromosome 7. Further region refinement revealed a region of 2 MB (Chr.7q34:139,681,371–141,507,822) (Figure 1) critical region shared among the affected individuals that might be harboring the plausible glaucoma causative gene. This region was not homozygous among any of the unaffected individuals. The novel locus 7q34 was flanked by D7S2202 (139,681,371‐139,881,718 cM:149.9) and D7S2513 (141,307,418‐141,507,822 cM:151.3).

Thirteen genes were present in 7q34 critical region (Table S3); thus, the affected family was subjected to whole‐exome sequencing for further analysis instead of targeted sequencing to allow a comprehensive search for rare variants within and beyond the linked region, and it resulted in the identification of 35,696 variants common among both individuals. One gene transmembrane protein 178B (*TMEM178B*) that was missing in the exome sequencing data was further analyzed by Sanger sequencing, based upon its presence in the novel identified locus, but no novel/known pathogenic mutations were identified. Nine homozygous variants were obtained after filtration of the exome sequencing data (Table S4) and were screened for segregation in the family. All the identified variants were present in the genes that were not localized in the identified 2 MB locus and were not segregating with the disease phenotype in the family.

#### 3.2.2. Identification of a Previously Unreported Variant p.Ser464Pro in *CYP1B1* in PCG07

In PCG07, we identified a previously unreported variant [NM_000104.4: c.1390T>C; p.Ser464Pro] (Table 2, Figure S4a). The variant was segregating with the disease phenotype in the family (Figure S2). This missense variant is missing in the Genome Aggregation Database (gnomAD), ClinVar database, and dbSNP. The variant has been reported once in Human Gene Mutation Database (HGMD), but no details about the variant or clinical manifestations have been provided. The variant is predicted as deleterious with score of 0.01 by SIFT and probably damaging with a score of 1.0 by Polyphen‐2, deleterious by MutationTaster (Table 3), and affects highly conserved amino‐acid residues of *CYP1B1* (Figure S4b). The variant has a negative *ΔΔ*G value of −2.03, which corresponds to the highly unstable protein structure (Table 3).

**Table 2:** *CYP1B1* mutations identified in the PCG families included in the study.

S. no.	Family ID	Affected members	Variant location (GRCh38)	Transcript ID	Nucleotide change	Amino acid change	dbSNP ID	MAF	Variant type	Variant status	Zygosity
1	PCG03	10	2:38071186–38071186G>T	NM_000104.4	c.1168C>A	p.Arg390Ser	rs148542782	0.00001637	Missense	Known	Homozygous
2	PCG04	11	2:38074520–38074521	NM_000104.4	c.868dup	p.Arg290Profs ^∗^37	rs587778875	0.0000186	Indel/frameshift	Known	Homozygous
3	PCG05	2	2:38075207C>T	NM_000104.4	c.182G>A	p.Gly61Glu	rs28936700	0.000208	Missense	Known	Homozygous
4	PCG06	2	2:38071023C>T	NM_000104.4	c.1331G>A	p.Arg444Gln	rs72549376	0.002	Missense	Known	Homozygous
5	PCG07	3	2:38070964A>G	NM_000104.4	c.1390T>C	p.Ser464Pro	NA	NA	Missense	Not reported	Homozygous
6	PCG08	4	2:38071185C>T	NM_000104.4	c.1169G>A	p.Arg390His	rs56010818	0.002	Missense	Known	Homozygous
7	PCG11	2	2:38074520–38074521	NM_000104.4	c.868dup	p.Arg290Profs ^∗^37	rs587778875	0.000026	Indel/frameshift	Known	Homozygous
			2:38074704–38074704C>T	NM_000104.4	c.685G>A	p.Glu229Lys	rs57865060	0.01038	Missense/hypomorphic allele	Known	Homozygous
8	PCG13	6	2:38071185C>T	NM_000104.4	c.1169G>A	p.Arg390His	rs56010818	0.002	Missense	Known	Homozygous

Abbreviations: c, coding DNA; MAF, minor allelic frequency; NA, not available; p, protein.

**Table 3:** In‐silico analysis of variants identified in the study.

Nucleotide change	Amino acid change	SIFT prediction	PolyPhen 2 prediction	Varsome	MutationTaster	I‐Mutant		Previously reported in the Pakistani population
						DDG (*ΔΔ*G) value (Kcal/mol)	Protein stability prediction	
c.1168C>A	p.Arg390Ser	Deleterious (0.00)	Probably damaging (1.0)	Pathogenic strong	Deleterious	−3.27	Decreased stability	Yes (17)
c.868dupC	p.Arg290Profs ^∗^37	—	—	Pathogenic	—	—	—	Yes (18, 19)
c.182G>A	p.Gly61Glu	Deleterious (0.00)	Probably damaging (1.0)	Pathogenic strong	Deleterious	−0.25	Decreased stability	Yes (20)
c.1331G>A	p.Arg444Gln	Deleterious (0.00)	Probably damaging (1.0)	Pathogenic	Deleterious	−1.51	Decreased stability	Yes (21)
c.1390T>C	p.Ser464Pro	Deleterious (0.01)	Probably damaging (1.0)	Pathogenic moderate	Deleterious	−2.03	Decreased stability	No
c.1169G>A	p.Arg390His	Deleterious (0.01)	Probably damaging (1.0)	Pathogenic	Deleterious	−2.81	Decreased stability	Yes (22)
c.685G>A	p.Glu229Lys	Tolerated (0.37)	Possibly damaging (0.522)	Benign	Benign	−1.03	Decreased stability	Yes (23)
c.1103G>A	p.Arg368His	Deleterious (0.00)	Probably damaging (1.0)	Conflicting	Deleterious	−1.70	Decreased stability	Yes (21)
c.1450C>T	p.Arg469Trp	Deleterious (0.00)	Probably damaging (1.0)	Pathogenic	Deleterious	−1.09	Decreased stability	Yes (21)
c.1325del	p.Pro442GlnfsTer15	—	—	Pathogenic/likely pathogenic	—	—	—	Yes (4)

#### 3.2.3. Structural Impact of p.Ser464Pro Variant

**Figure 2:**
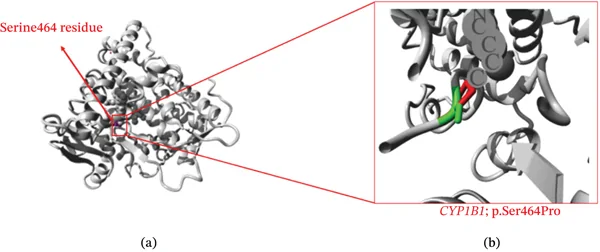
In‐silico protein prediction of variant p.Ser464Pro protein structure. (a) The position of the amino acid residue Ser464 in the protein structure (magenta color). (b) The zoomed‐in version of change in protein structure due to Ser464Pro variant (wild type is colored green, whereas the mutant is colored red).

**Figure 3:**
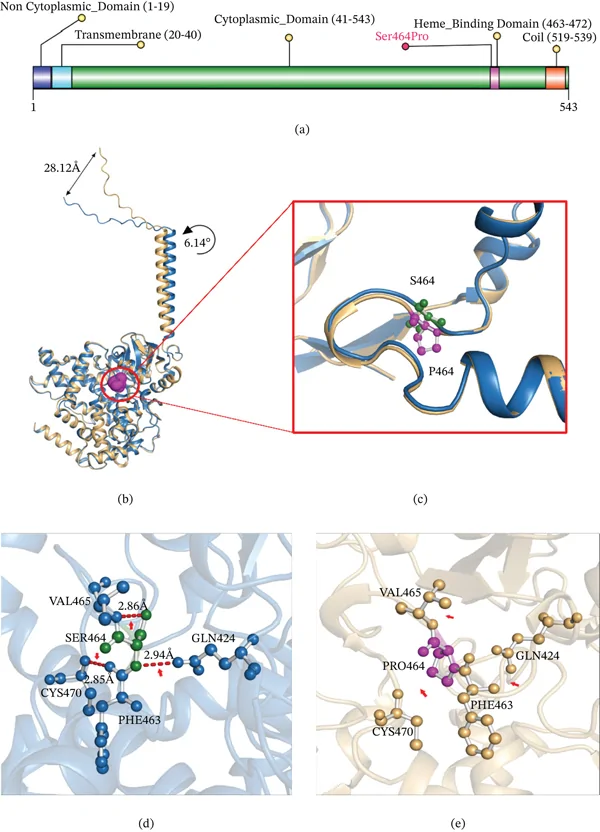
In‐silico mutational analysis of CYP1B1 protein. (a) Schematic representation of full‐length CYP1B1 protein showing domains of full‐length protein (mutant Ser464Pro is colored red). (b) Superimposed 3D structure of wild‐type and Ser464Pro mutant CYP1B1 proteins. Wild type protein is represented by sky blue color and mutant protein by light orange color indicating that mutant protein experienced a structural deformation of 6.14° of helix, whereas loop deviation distance is 28.12 Å. (c) Zoom‐in picture for close‐up view of the Ser464Pro represented by forest green color and magenta color, respectively. (d,e) Comparison of Residues 464 in wild‐type and mutant CYP1B1 indicating the Serine 464 from wild type showed two hydrogen bonds with surrounding residues, whereas mutant Proline 464 disrupts these interactions, indicating local structural alteration.

HOPE predicted the position of mutant residue (Figure 2) and the InterPro analysis indicated that Ser464 resides on heme‐binding domain (Figure 3a), and proline residues (a known helix breaker) introduce a kink and affect the structure of mutant protein. The structural deviation of helix is 16.4° after mutation, and loop deviation measurement is 28.18 Å between both structures (Figure 3b,c). Moreover, in the wild‐type structure, serine at Position 464 forms two hydrogen bonds, with lengths of approximately 2.94 and 2.86 Å (Figure 3d), and the variant results in disruption of both bonds (Figure 3e). Interestingly, the Ser464Pro also disrupts one hydrogen bond (Figure 3e) of length 2.85 Å (Figure 3c) of the adjacent phenylalanine residue at Position 463.

#### 3.2.4. Identification of Recurrent Mutations in *CYP1B1* in Seven Families

Out of eight families linked to *CYP1B1*, seven were linked to known homozygous mutations among which four were missense [NM_000104.4: c.1331G>A; p.Arg444Gln, c.1169G>A; p.Arg390His, c.1168C>A; p.Arg390Ser, and c.182G>A; p.Gly61Glu], one was a frameshift [NM_000104.4: c.868dup; p.Arg290Profs ^∗^37], and one hypomorphic allele [c.658G>A; p.Glu229Lys] (Table 2), and all the identified variants were segeregating with the phenotype in an automsomal recessive inheretance pattern (Figure S2). Two unrelated consangunieous families (PCG08 and PCG13) harbored a frequent mutation, [p.Arg390His; rs56010828] (Table 3), homozygouly (Figure S2). In the family PCG08, all the affected individuals were homozygous for p.Arg390His, whereas eight uneffected individuals were carriers of the mutation (Figure S2).

In PCG13 all the affected individuals were homozygous for the mutation whereas all the unaffected members were carrier of the mutation (Figure S2). A previously known duplication mutation c.868dup, causing frameshift is predicted to cause premature truncation [p.Arg290Profs ^∗^37; rs587778875] of the protein (Tables 2 and 3), was identified homozygously in five affected members of PCG04 and two affected members of PCG11 (Figure 1). Additionally, a known hypomorphic allele [p.Glu229Lys; rs57865060] was also identified in both affected individuals of PCG11 (Table 2). Family PCG06 had a reported [p.Arg444Gln; rs72549376] mutation (Table 2) considered to be pathogenic by prediction tools (Table 3). Both the affected individuals were homozygous for the mutation whereas two unaffected members were carrier of the mutation. In PCG03 and PCG05, known mutations [p.Arg390Ser; rs148542782], and [p.Gly61Glu; rs28936700] (Table 2) were identified, respectively.

#### 3.2.5. Identification of Recurrent Mutations in *CYP1B1* in Three Trios Families

Three recurrent mutations including two missense: [NM_000104.4: c. 1103G>A; p.Arg368His, and c.1405C>T; p.Arg469Trp], and a frameshift: [NM_000104.4: c.1325del; p.Pro442GlnfsTer15] were identified in three PCG families from the trios analyses (Table S5). In a trios family PCGT01, two pathogenic variants (Table S5) [p.Arg368His; rs78204362] and [p.Arg390His; rs56010818] were present heterozygously (Table S5). In PCGT02, we identified a known homozygous variation [p.Arg469Trp; rs28936701] (Table S5). In the trios PCGT03, a known homozygous deletion mutation [p.Pro442GlnfsTer15; rs770799584] was identified (Table S5).

## 4. Discussion

PCG is an inherited disorder, with high prevalence in regions with high rates of consanguineous marriages [17]. In Pakistan, approximately 63% marriages are reported to be between first cousins [7], and this higher ratio of consanguinity correlates with higher rate of inherited disorders [18]. PCG is a genetically heterogenous disease that has previously been linked to five loci GLC3A‐E [12, 19]. In this study, we report a plausible novel locus at Chromosome 7q34 (139,681,371–141,507,822). The 7q34 locus has been subjected to extensive investigations as the region is involved in various tumors including T‐lymphoblastic leukemia [20], myeloid malignancies [21], and 13 other malignant tumors [22]. The region 7q34 has also been reported to cause developmental delays, hearing loss, growth retardation [23], motor neuron degeneration [24], and Alzheimer′s disease [25]. Despite the involvement of locus in various disorders, none of the genes in the locus have been reported to cause PCG phenotypes. However, the plausible novel locus identified in this study harbors solute carrier family 37 member 3 (*SLC37A3*; OMIM:619137) (chr7: 140,293693–140,404,433) and a splice site mutation [c.521+1G>A] in this gene has been reported to be linked with autosomal retinitis pigmentosa (RP) [26]. Gopinath et al. [24] have also reported Chromosome 7q34–q36 as a novel locus for motor neuron disease (MND) and analyzed cycling dependent kinase 5 (*CDK5*; OMIM: 123831) residing in the candidate interval, but no mutations were identified in the gene.

Despite using high‐throughput techniques, no novel or known variants were identified in the PCG02, suggesting that the disease causative variants might be residing in the nonexonic or deep genomic regions. Rauf et al. [27] also faced similar challenge when exome sequencing failed to identify any causative variant in 10 affected individuals belonging to four unrelated PCG families of Pakistani origin. Similarly, Al‐Saei et al. [28] were also unable to solve 29 PCG families after employing WES. A limitation of the present study is the small size of the pedigree, with only two affected individuals available for genetic analysis. Consequently, the linkage evidence supporting the identified 2 MB homozygous region is not statistically robust; therefore, this region is considered a plausible novel locus rather than a confirmed novel PCG locus.

Although various strategies of gene identification were employed in this study, efforts remained unsuccessful; rather a genomic region 7q34 (139,681,371–141,507,822) was attributed to the diseased phenotype in the family. Further analysis with whole‐genomic sequencing of the patients might help in the identification of any insertion, deletion, or substitution in the deep intronic region that may be associated with PCG. Mutations in deep intronic regions are a likely cause for different diseases by altering gene expression levels through changes in splicing regulatory elements or activation of noncanonical splice sites. These mutations can also disrupt noncoding RNA genes and transcription regulatory motifs [29]. Studies have shown that intronic mutations in regions of *CYP1B1* may also lead to PCG [9]. Similarly, five different mutations in the intronic regions of myocilin gene have been identified associated with primary open‐angle glaucoma (POAG) [30].

Along with a plausible novel locus, we have also identified previously unreported and recurrent mutations in *CYP1B1*. Mutated *CYP1B1* is one of the major genes implicated in PCG in the Pakistani population (34.6%) [18] and globally [31]. The gene is a member of Cytochrome P450 family of genes that are responsible for the detoxification of exogenous molecules like polycyclic aromatic hydrocarbons and endogenous molecules like estradiol. *CYP1B1* is primarily expressed in ciliary body, iris, TM, and retina, and plays a role in processes involved in eye development [32].

To date, more than 250 pathogenic mutations in *CYP1B1* have been associated with PCG [17, 33]. We identified a previously unreported missense mutation c.1390T>C [p.Ser464Pro] in the Pakistani population that was not found in any online database except HGMD. Kaur et al. [34] mentioned the variant in Indian population, and no additional data were provided about the variant. The Serine residue at 464 resides in the heme‐binding domain of CYP1B1, which is essential for monooxygenase activity of the enzyme [35]. The substitution of serine with proline affects conformational changes that might affect enzyme function by compromising the substrate binding or electron transfer efficiency, leading to reduced catalytic activity. This suggests that even a single‐point mutation residing in functionally important regions of proteins has harmful effects. Previously, researchers have demonstrated that a point mutation p.Gly466Asp identified in PCG patients had most disruptive effect on substrate binding region of CYP1B1 enzyme, with distorted substrate‐binding pocket [36].

No clinical data for the family having the variant was given in available databases; therefore, our clinical findings could not be compared. However, compared with the clinical manifestations of PCG families carrying other mutations in current study, it was observed that patients carrying p.Ser464Pro had less severe symptoms due to early preventative measures, thereby resulting in preserved vision, whereas the patient without the early preventative measures was completely blind.

We also identified p.Arg390His in our cohort, which is one of the most common mutations in Asian populations [37–40], and is considered as the founder mutation in the Pakistani population (45%) [18]. The mutation p.Arg390His was identified in 27% of cases in current cohort (three families), which is consistent with previously reported frequencies in the region. The mutation p.Arg368His, which is the second most prevalent mutation in the Pakistani population (5.8%) [18], was identified in only one trios family as a compound heterozygote along with p.Arg390His. The variant p.Arg368His has been reported as compound heterozygote with pArg390Cys in the Indian population as well [41]. Micheal et al. [42] reported compound heterozygous mutations (p.Arg390His/p.Ser464Thr; p.Arg368His/p.Arg444 ^∗^) in two PCG families of Pakistani origin. We further identified p.Gly61Glu that has been previously reported in a single conganuineous Pakistani family with coexistance of PCG and JOAG in two subsequent generations [43], but our family had presentation of PCG only. The variant has also been idenitfied in PCG families of other populations including Iran [44], Iraq [45], Morroco [46], and Saudi Arabia [47].

We also identified p.Arg290Profs ^∗^37 in 18% of cases (two families). The mutation was first identified as a novel mutation in the Pakistani population in our previous cohort [42]. Among the two families with p.Arg290Profs ^∗^37, one family (PCG11) also had a hypomorphic allele p.Glu229Lys that has been previously reported as a pathogenic mutation in the Pakistani population [40, 48–49]; however, in our study, the in‐silico tools predicted the mutation to be benign or tolerated with two tools; Polyphen‐2 predicting a possibly damaging effect and I‐Mutantv2.0 suggesting a potential reduction in protein stability. Therefore, functional studies along with in‐silico predictions are essential to enhance the understanding of the underlying molecular mechanisms for p.Glu229Lys. Studies have reported that p.Glu229Lys reduces the CYP1B1 enzymatic activity by 20%, leading to classification of mutation as a hypomorphic allele with a decreased function, and it has been proposed that this mutation could function as a risk allele that might lead to glaucoma development in the presence of modifier genes [50, 51].

Clinical evaluation of the affected individuals with homozygous variants in *CYP1B1* revealed interfamilial and intrafamilial variability in clinical manifestations of PCG. This clinical variability might be indication of the incomplete penetrance and variable expressivity of *CYP1B1* alleles. This phenomenon has previously been observed in Saudi Arabian [4] and Pakistani [52] consanguineous PCG families. The incomplete penetrance might be the result of epigenetic and other environmental factors [52]. The presence of a modifier gene has also been proposed, although no such factor has been reported yet [4]. The identification of genetic modifiers that suppress the Mendelian disease mutant phenotypes can enhance our understanding of mechanisms by which organisms mitigate the effects of pathogenic mutations [52–54]. Moreover, the digenic inheritance such as concurrent variants in *CYP1B1* and *TEK* or *MYOC* might also complicate the variability of clinical manifestations in PCG [55]. Chakrabarti et al. [56] reported the digenic inheritance of *TEK* and *CYP1B1* mutations and observed that both genes interact genetically as well as physically with each other. The digenic mode of inheritance was also reported in Northern Indian JOAG family in which the affected individuals with cosegregation of mutations in *MYOC* and *LTBP2* showed severe phenotypes [57]. The identification of digenic inheritance is necessary for understanding the spectrum of genetic factors in PCG and explaining the associated clinical heterogeneity.

## 5. Conclusion

This study identified *CYP1B1* mutations in 88.9% of the studied Pakistani PCG families, confirming it as the most frequently mutated gene in this cohort. The findings highlight the major contribution of *CYP1B1* to the genetic etiology of PCG in the Pakistani population. Given its high mutation frequency, *CYP1B1* should be prioritized in population‐specific genetic screening and carrier‐testing strategies for PCG in Pakistan. The high prevalence of *CYP1B1*‐associated PCG also supports exploring its potential incorporation into targeted noninvasive prenatal testing (NIPT) approaches for families at increased genetic risk, subject to further validation. These findings provide a basis for developing targeted genetic screening and early diagnostic strategies for PCG in the Pakistani population.

## Funding

This study was supported by Higher Education Commission, Pakistan (10.13039/501100004681; 20‐17501/NRPU/R&D/HEC/2021).

## Ethics Statement

This study was approved by the Research Ethical Committee (REC) of Pak‐Austria Fachhochschule: Institute of Applied Science and Technology (Approval No. PAF‐IAST/2021/08).

## Consent

Informed consent was obtained from all individual participants included in the study. The authors affirm that human research participants provided informed consent for publication of the images in Figure S1.

## Conflicts of Interest

The authors declare no conflicts of interest.

## Supporting information


**Supporting Information** Additional supporting information can be found online in the Supporting Information section. Figure S1: Clinical analyses of the two affected members in the Pakistani PCG family. Figure S2: Pedigrees of eight consanguineous PCG families and three trios families of Pakistani origin with segregation of disease‐causing variants in *CYP1B1*. Figure S3: HomozygosityMapper plot of the congenital glaucoma family. Figure S4: An electropherogram of the DNA sequence with new single nucleotide variant (SNV) (p.Ser464Pro) and multiple sequence alignment of CYP1B1 protein. Table S1: Chromosome 7 microsatellite markers used for region refinement and carrier status identification in PCG02 family. Table S2: Homozygous regions obtained on Chromosomes 3, 7, and 15 by homozygosity mapping. Table S3: List of genes in novel locus on Chromosome 7. Table S4: Homozygous variants detected by exome sequencing and analyzed for segregation analysis in PCG02 family. Table S5: Known mutations identified in *CYP1B1* the PCG trios familial cases included in the current study cohort.

## Data Availability

The data that support this study are available from the corresponding author upon reasonable request.
