# Supplementary material for: Uncovering the Novel Genetic Determinants of Primary Congenital Glaucoma in Pakistani Families
Source: Hum Mutat. 2026 Sep 18;2026:1806353. doi: 10.1155/humu/1806353 (PMC13586781; doi:10.1155/humu/1806353)
Supplement: Supplementary file 1 [file HUMU-2026-1806353-s001.docx]

**Supplementary Figure 1: Clinical analyses of the two affected members in the Pakistani PCG family** **i)** Shows an opaque cornea which bulges out due to elevated levels of IOP in individual II:4**. ii**) Shows an opaque cornea of individual II:5, and also bulging of the eye. Both PCG patients were completely blind, **iii)** showing the B-scans done for II:4. The B-scan of the right eye shows an integral haze with a total freely mobile fibrotic posterior vitreous detachment. The optic nerve head shows a markedly deep excavated cup indicated with an arrow. The B-scan of the left eye also shows a deep excavated cup (indicated with an arrow), but the patient had normal tomographs.

**Supplementary Figure 2. Pedigrees of eight consanguineous PCG families and three trios families of Pakistani origin with segregation of disease-causing variants in *CYP1B1*.** Roman numerals represent the generation numbers. Filled symbols (Squares and circles) are showing the affected individuals while the empty square and circle are showing the unaffected individuals. A double line connecting the two individuals represents the consanguineous marriage. Arrow represents the probands in the respective families. Genotypes of the enrolled individuals are written below the individual generation numbers. ‘+’ indicates the wild type allele, ‘M’ indicates the mutant allele. +/+ represents the individuals with homozygous ancestral genotype, +/M indicates the individuals with one wild type allele and the mutant allele, and M/M indicates the individuals carrying homozygous mutant alleles.

**Supplementary Figure 3.** **Homozygosity Mapper plot of the congenital glaucoma family**; on the x-axis, we have number of chromosomes, on the y axis homozygosity scores are shown. The threshold value for homozygous region to be significant was 0.8 and for this congenital glaucoma family we obtained three homozygous regions that crossed the threshold level. After segregation analysis of all these regions by using microsatellite markers, in family members, the regions on chr. 3 and 15 were excluded thus identifying one 10 MB true homozygous region on chr. 7 as it segregated with phenotype in the family.

**Supplementary Figure 4. An electropherogram of the DNA sequence with new single nucleotide variant (SNV) (p.Ser464Pro) and multiple sequence alignment of CYP1B1 protein.** **a)** The variant sequence (middle panel) is shown along with the wild type homozygous sequence (upper panel), and heterozygous sequence (lower panel). Arrows represent the position at which the change occurs. **b)** ClustalW alignment of CYP1B1 orthologs show the conservation of amino acid residue at position 464 among various species.

**Supplementary Table 1. Chromosome 7 microsatellite markers used for region refinement and carrier status identification in PCG02 family**

| Marker | Location on chromosome in bp | | cM |
| --- | --- | --- | --- |
|  | **Start** | **End** |  |
| D7S495 | 137,907,609 | 138,107,974 | 145.3 |
| rs10435432 | 138,493,926 | 138,494,426 |  |
| rs451054 | 138,435,565 | 138,436,065 |  |
| rs3734938 | 138,423,565 | 138,424,296 |  |
| D7S2202 | 139,681,371 | 139,881,718 | 149.9 |
| D7S2513 | 141,307,418 | 141,507,822 | 151.3 |
| D7S794 | 143,623,640 | 143,823,942 | 155.1 |
| D7S676 | 143,982,666 | 144,183,018 | 156.1 |
| D7S688 | 148,443,795 | 148,644,059 | 159.5 |

bp; base pair, cM; centimorgan

| Chromosome | Size of regions | Location of regions |
| --- | --- | --- |
| 3 | 15 MB | 43,801,895-58,931,816 |
| 7 | 10 MB | 138,534,971-147,937,132 |
| 15 | 1.5 MB | 56,112,714-58,070,608 |
| 15 | 2 MB | 60,108,779-61,437,557 |
| 15 | 7 MB | 71,679,959-78,435,527 |

**Supplementary Table 2.** **Homozygous** **regions obtained on chromosome 3, 7 and 15 by homozygosity mapping**

**Supplementary Table 3.** **List of genes in novel locus on chromosome 7**

| **S#** | **Gene Name** | **Function** |
| --- | --- | --- |
| 1 | *HIPK2* | Functions as a corepressor and coactivator |
| 2 | *TBXAS1* | Monooxygenase activity |
| 3 | *PARP12* | Controls protein translation and NF-kB signaling |
| 4 | *KDM7A* | Play a role in Histone code and brain development |
| 5 | *SLC37A3* | Has inorganic phosphate antiporter activity |
| 6 | *RAB19* | Has GTPase activity |
| 7 | *MKRN1* | Acts as negative regulator of telomerase |
| 8 | *DENND2A* | Helps in activation of RAB8A and RAB9B |
| 9 | *NDUFB2* | Accessory subunit of the mitochondrial membrane respiratory chain NADH dehydrogenase involved in catalysis |
| 10 | *ADCK2* | Protein kinase activity |
| 11 | *BRAF* | Involve in transduction of mitogenic signals from the cell membrane to the nucleus |
| 12 | *MRPS33* | Helps in protein synthesis within the mitochondrion |
| 13 | *TMEM178B* | Negative regulator of excessive osteoclast (OC) |

**Supplementary Table 4. Homozygous variants detected by exome sequencing and analyzed for segregation analysis in PCG02 family**

| **Gene name** | **Chromosome** | **Variant** | **SNP Frequency** | **PhyloP** | **Grantham** | **CADD Score** | **SNP/ Unreported**  **Variant** |
| --- | --- | --- | --- | --- | --- | --- | --- |
| *SH3PXD2B* | 5 | 2629del; p.Phe876Lysfs*16 | N/A | 4.35 | 1000 | 35 | Unreported |
| *CACNA1D* | 3 | 2474T>C; p.Ile805Thr | 0.00000344 | 4.27 | 89 | 19.38 | rs147933585 |
| *CILP* | 12 | 217G>A; p.Arg73Trp | 0.00005 | 3.80 | 101 | 25.5 | rs368935809 |
| *ANKDD1A* | 15 | 1096C>T; p.Arg366Cys | 0.000965 | 2.5 | 180 | 0.538 | rs144426862 |
| *LRRC8E* | 19 | 580G>C; p.Gly194Arg | 0.000024 | 2.33 | 125 | 14.21 | rs748956991 |
| *CSPG4* | 15 | 4813G>A; p.Leu1605Phe | 0.000368 | 2.22 | 24 | 22.5 | rs548700280 |
| *CLRN3* | 10 | 532C>T; p.Gly178Arg | 0.00243 | 1.89 | 125 | 21.3 | rs375816600 |
| *ALS2CL* | 3 | 2843T>G; p.His948Arg | 0.000012 | 0.46 | 77 | 14.92 | rs774761469 |
| *TRAIP* | 3 | 898C>T; p.Glu300Lys | 0.000000693 | 2.83 | 56 | 23.2 | rs143310707 |

| **S. No.** | **Trios ID** | **Variant location**  **(GRCh38)** | **Transcript ID** | **Nucleotide change** | **Amino acid change** | **dbSNP ID** | **MAF** | **Variant type** | **Variant status** | **Zygosity** |
| --- | --- | --- | --- | --- | --- | --- | --- | --- | --- | --- |
| **1** | **PCGT01** | 2:38071251 C>T | NM_000104.4 | c.1103G>A | p.Arg368His | rs78204362 | 0.003119 | Missense | Known | Heterozygous |
|  |  | 2:38071185 C>T | NM_000104.4 | c.1169 G>A | p.Arg390His | rs56010818 | 0.002 | Missense | Known | Heterozygous |
| **2** | **PCGT02** | 2:38070949 G>A | NM_000104.4 | c.1450C>T | p.Arg469Trp | rs28936701 | 0.00004151 | Missense | Known | Homozygous |
| **3** | **PCGT03** | 2:38071029 | NM_000104.4 | c.1325del | p.Pro442GlnfsTer15 | rs770799584 | 0.000004 | Indel/Frameshift | Known | Homozygous |

**Supplementary Table 5.** **Known** **mutations identified in *CYP1B1* the PCG trios familial cases included in the current study cohort**

Id; Identity, c; Coding DNA, p; Protein, db; Database, MAF: Minor allelic frequency
